# Supplementary material for: High-resolution spectroscopy of single nuclear spins via sequential weak measurements
Source: Nat Commun. 2019 Feb 5;10:594. doi: 10.1038/s41467-019-08544-z (PMC6363762; doi:10.1038/s41467-019-08544-z)
Supplement: Supplementary file 1 — Supplementary Information [file 41467_2019_8544_MOESM1_ESM.pdf]

# Supplementary Information

# for

"High-resolution spectroscopy of single nuclear spins via sequential weak measurements"

by Pfender, M. et al.

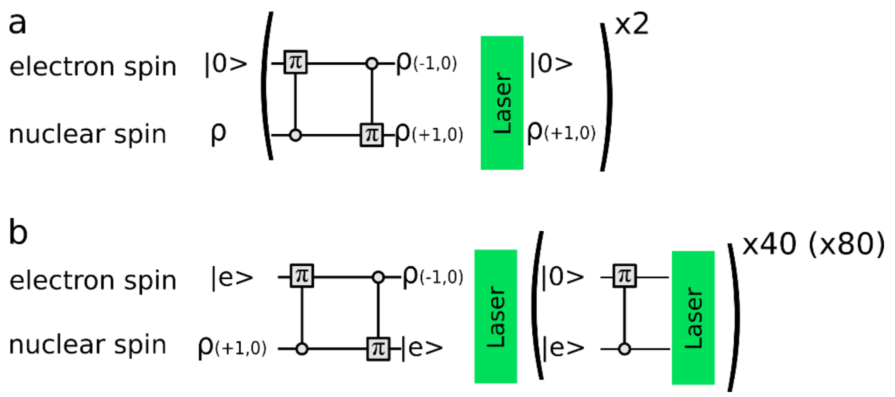


**Supplementary Figure 1: Pulse sequences for repetitive readout.** (a) shows the initialization of the $^{14}N$ nuclear spin. Starting with an initialized electron and unpolarized nuclear spin, a SWAP gate consisting of two consecutive CNOT gates on the electron and nuclear spin, respectively, is performed. This initializes the $^{14}N$ nuclear spin in the desired $m_I = 0$ and $m_I = +1$ submanifold. A laser pulse then reinitializes the electron spin, leaving the nuclear spin state untouched. The whole sequence is performed twice. (b) After the measurement on the target spin, the electron spin state is read out via repetitive readout of the $^{14}N$ nuclear spin. An arbitrary electron state $|e>$ is transferred onto the $m_I = 0$ and $m_I = +1$ nuclear spin manifold by a SWAP gate. Afterwards, the nuclear spin state can be transferred onto the electron spin, and read out. Due to the stability of the $^{14}N$ state under optical illumination, this can be done repetitively (40 or 80 times in this work), effectively increasing the number of photons collected for one measurement on the target spin.

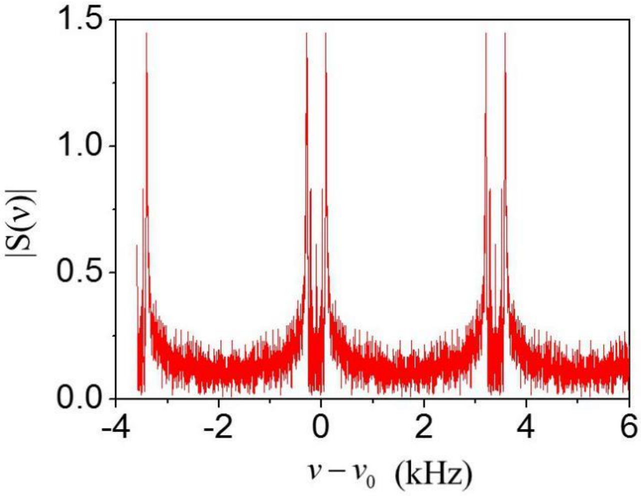


**Supplementary Figure 2: Photon shot noise fluctuation of the correlation spectrum.** The fluctuation due to photon shot noises is about 0.2. The experimental condition and parameters are the same as in Fig.4(a) in the main text. Source data are provided as a Source Data file.

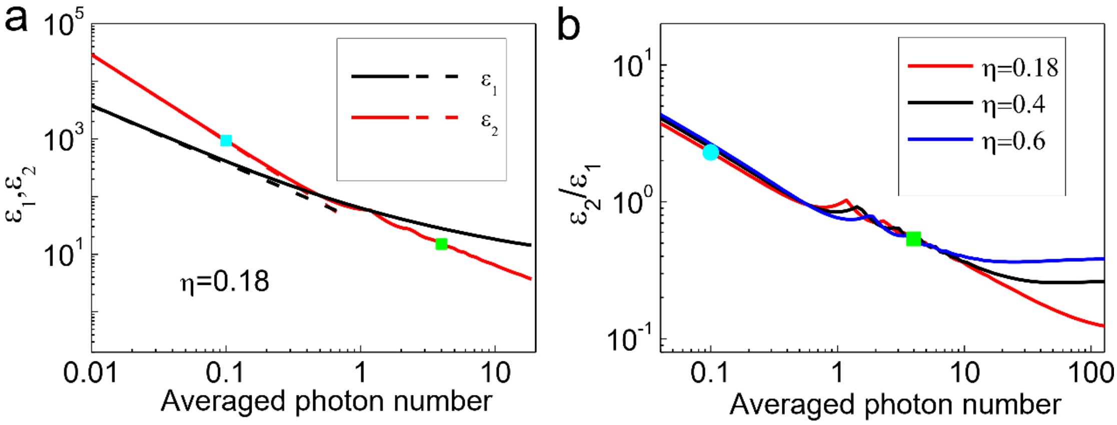


**Supplementary Figure 3: Comparison between the methods for constructing correlation of weak measurements from photon counts.** (a) The fluctuation of correlations that are reconstructed with the two different methods (see Supplementary Note 5), plotted as a function of $\bar{n}$. The photon count contrast is fixed to be 0.18. The solid line denotes the exact result while the dashed line denotes the approximate result at the limit of the low photon number. (b) The ratio between the fluctuations of the correlations obtained by the two methods, plot as a function of $\bar{n}$. The curves of different colors correspond to different photon count contrasts $\eta$ as indicated in the legend. The parameters corresponding to Figs. 2 & 3 in the main text are marked in the figures by the Cyan symbols while those corresponding to Fig.4 are marked by the Green symbols. Source data are provided as a Source Data file.

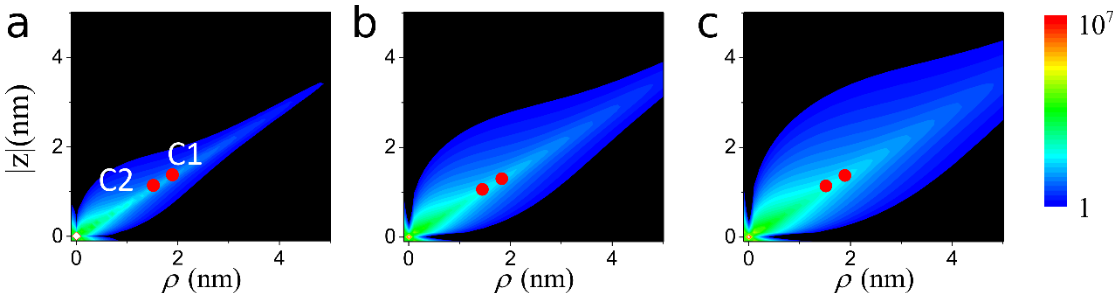


**Supplementary Figure 4: Spatial range of sensing single nuclear spins**. The color contour is the signal-to-noise ratio of the resonance peak of the correlation spectrum of a $^{13}$C nuclear spin at different locations, given by the cylindrical coordinates (radius $\rho$ from the NV axis and the z coordinate along the NV axis). The interaction time $t_I$ between the NV center electron spin and the target nuclear spin is determined by the number of DD control pulses $N_p$. The effective duration for extra dephasing is $\tau_{eff} \approx 600$ μs. The other parameters are the same as in Figs. 4 in the main text. (a), (b), and (c): $N_p = 100$, 200 and 300 in turn. The red spots are the locations of the nuclear spins C1 and C2 observed in Fig. 4 of the main text. For all the figures, the number of measurement cycles is $M = 3 \times 10^7$. Source data are provided as a Source Data file.

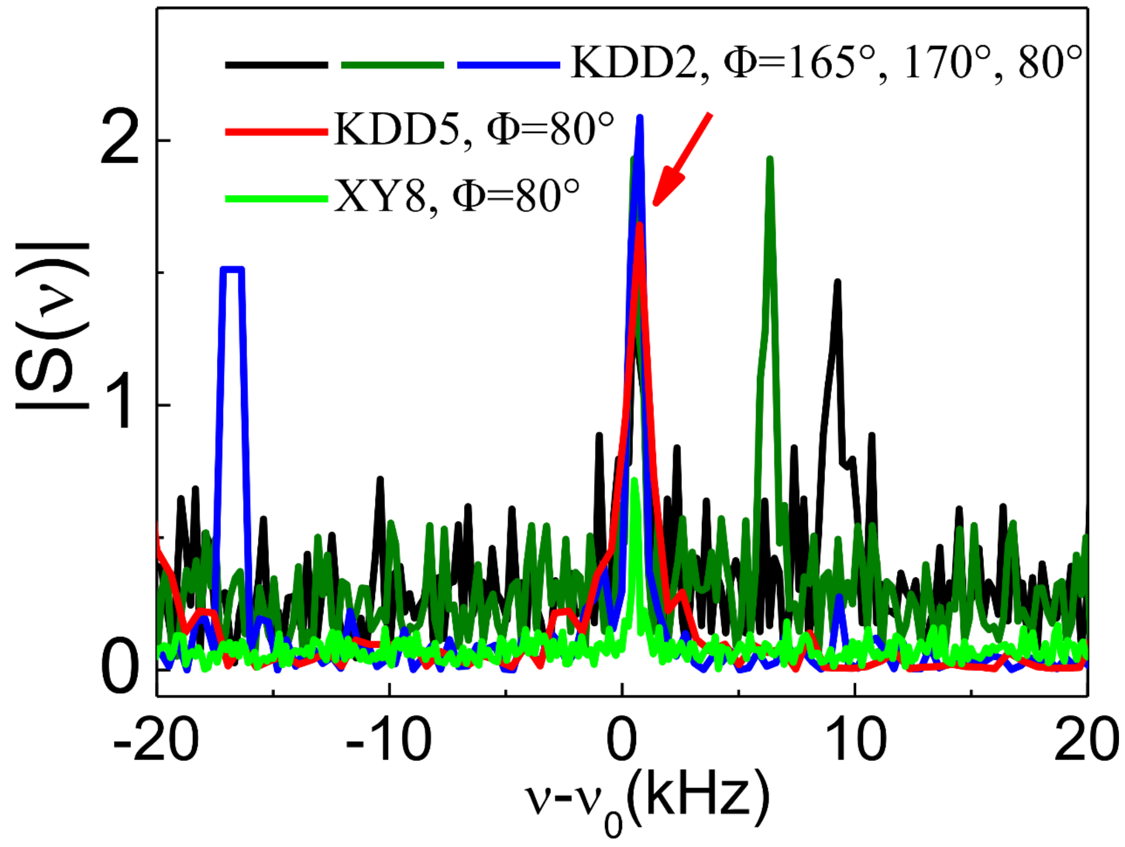


**Supplementary Figure 5: Determination of the hyperfine coupling.** Correlation spectrum $|S(\nu)|$ for various interaction time $t_{\rm I}$ and cycle period $t_{\rm c}$. For XY8, KDD2 and KDD5, $t_{\rm I} = 1.45920$ μs, $7.29600$ μs, and $18.24000$ μs in turn. The Larmor frequency is $\nu_0 \approx 2.743189$ MHz (the same as in Figs. 2-4 of the main text). Source data are provided as a Source Data file.

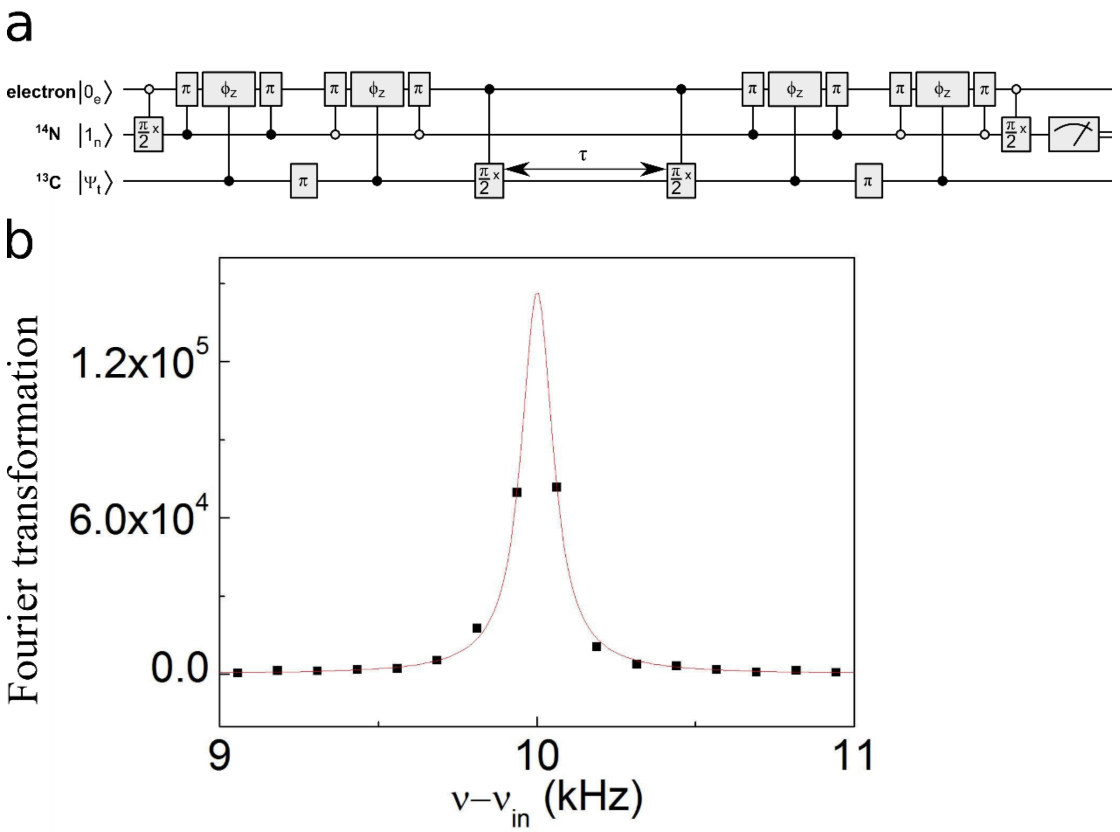


**Supplementary Figure 6: High-precision measurement of the bare Larmor frequency of $^{13}$C nuclear spins.** (a) Measurement sequence to determine the bare $^{13}$C Larmor frequency by employing a hybrid spin sensor (see Ref. [1]). The sequence consists of four phase accumulation parts (denoted by $\Phi_z(\tau)$), separated by storage of the accumulated phase on the nitrogen nuclear spin, as well as radio-frequency manipulation of the $^{13}$C spins. During the manipulation, the NV centre electron spin is in the $m_S = 0$ state (hence no hyperfine interaction on the nuclear spins). The measurement is performed by varying the time $\tau$ between the two $\frac{\pi}{2}$ pulses on the $^{13}$C spins, performing an FID measurement. (b) Fourier transformation of the FID signal for measuring the bare Larmor frequency with the method in Ref. [1]. Source data are provided as a Source Data file.

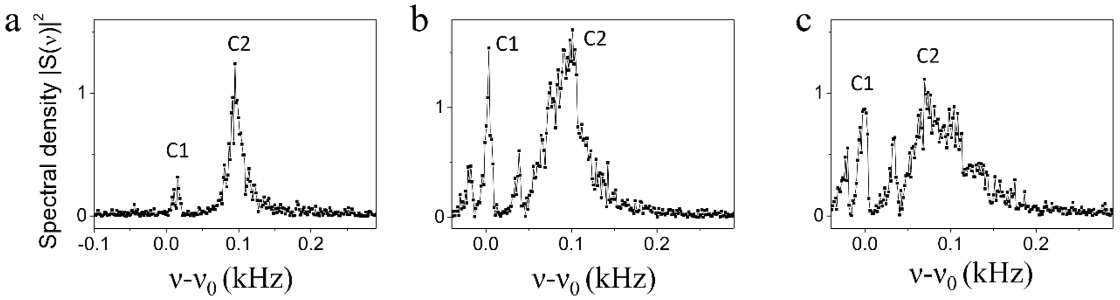


**Supplementary Figure 7 | High-resolution spectroscopy of single nuclear spins (Data1).** Fourier transform of the correlation constructed from Data1. The electron spin is repetitively read out for 40 times in each measurement cycle. The number of DD pulses is (a) $N_{\mathrm{p}} = 100$, (b) $N_{\mathrm{p}} = 200$, and (c) $N_{\mathrm{p}} = 300$. The bare Larmor frequency is measured to be $\nu_0 = 2.740134 \pm 0.39$ Hz. The number of time points for Fourier transform is $N_{\mathrm{FT}} = 2000$. Source data are provided as a Source Data file.

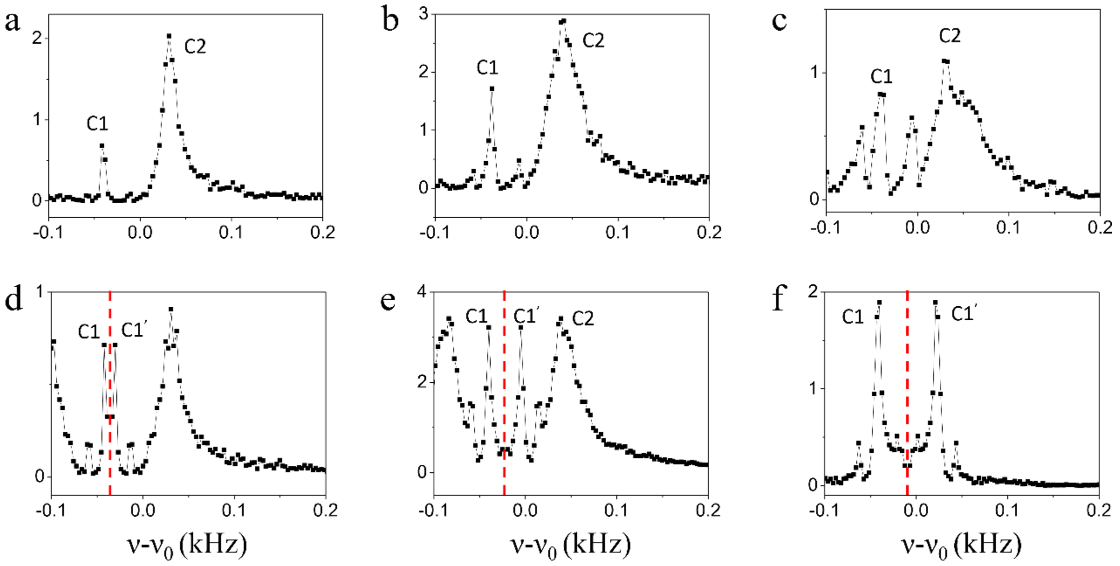


**Supplementary Figure 8 | High-resolution spectroscopy of single nuclear spins (Data2).** Fourier transform of the correlations constructed from Data2. In each measurement cycle the electron spin is repetitively read out for 40 times in (a-c) and 80 times in (d-f). The number of DD pulses is (a/d) $N_{\mathrm{p}} = 100$, (b/e) $N_{\mathrm{p}} = 200$, and (c/f) $N_{\mathrm{p}} = 300$. The red line at $2\pi \nu t_{\mathrm{c}} = 0 \bmod 2\pi$ is the symmetric line. The number of time points for Fourier transform is $N_{\mathrm{FT}} = 1000$. Source data are provided as a Source Data file.

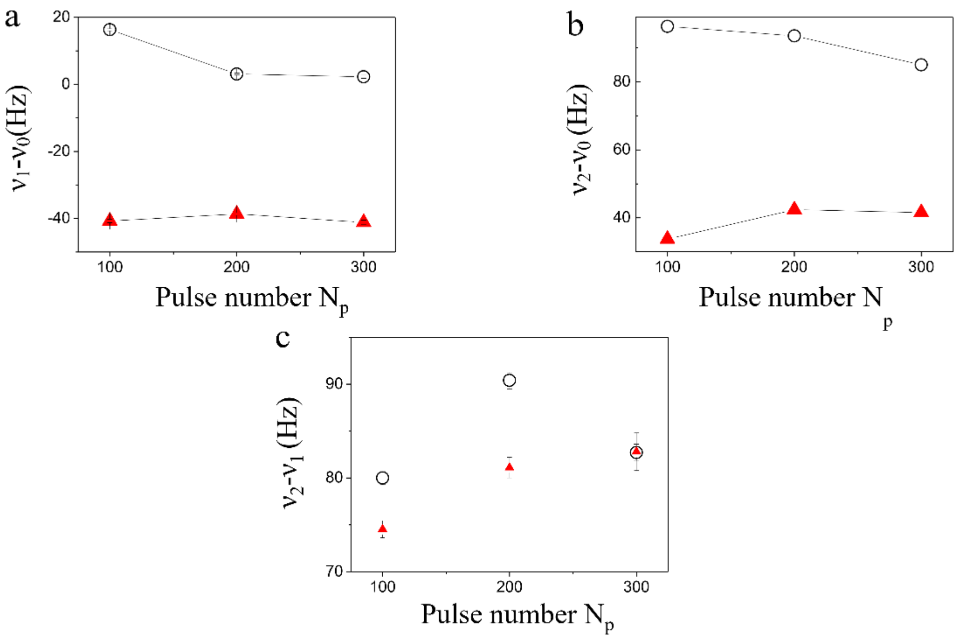


**Supplementary Figure 9 | Shift of the bare Larmor frequency between the two data sets**. (a) Frequency of C1, (b) frequency of C2, and (c) frequency difference between C1 and C2, obtained from Data1 (black circles) and Data2 (red triangles), as functions of the DD pulse number. Source data are provided as a Source Data file.

## Supplementary Note 1: $^{14}$N nuclear spin assisted readout of NV centre electron spin

When detecting spins via the autocorrelation of subsequent measurements, the readout efficiency of the electron spin is a critical parameter. It can be increased by transferring the electron spin state to the $^{14}$N nuclear spin, which can be read out repetitively in a non-demolition way[2,3]. Since only the $m_S = 0$ and $m_S = -1$ spin manifold is used, the $^{14}$N nuclear spin needs to be initialized into a sub-manifold consisting of two eigenstates, in our case $m_I = 0$ and $m_I = +1$. This is done by two conditional $\pi$ rotation of the electron and nuclear spin, which constitutes a SWAP gate between the NV electron spin and the $^{14}$N nuclear spin. Subsequently, the NV electron spin is reinitialized (see Supplementary Fig. 1 (a)). Due to insufficient initialization of the combined NV electron spin and charge state (around 70% $NV^-$ and 30% $NV^0$)[4], the procedure is done twice.

After the measurement, the electron spin state is again transferred onto the nuclear spin (see Supplementary Fig. 1 (b)) by a SWAP gate. Owing to the stability of the $^{14}$N spin state during optical excitation, the nuclear spin state can be transferred repetitively to the electron spin state, and read out (40 times in our experiments). This method is used for Fig. 4 in the main text.

## Supplementary Note 2: Correlation of sequential weak measurement - formalism

### *2.1. Control phase gate by dynamical decoupling sequence*

Each measurement cycle includes four steps (see Fig. 1g in the main text):

1. the electron spin is initialized to the x state (by initialization to the z direction state and then rotation by a $(-\pi/2)_{\mathrm{y}}$ pulse);
2. evolution under DD control for a period of $t_{\mathrm{I}}$;
3. free evolution for a waiting time $t_{\mathrm{read}}$;
4. Measurement of the electron spin y component (realized by a rotation by a $(\pi/2)_{\mathrm{x}}$ pulse and then measurement of the z component via photoluminescence).

The NV center spin $\hat{\boldsymbol{S}}$ and the nuclear spin-1/2 $\hat{\boldsymbol{I}}$ have the interaction

$$\hat{H} = |+\rangle\langle+| \otimes \boldsymbol{\nu}_+ \cdot \hat{\boldsymbol{I}} + |-\rangle\langle-| \otimes \boldsymbol{\nu}_- \cdot \hat{\boldsymbol{I}}, \tag{1}$$

where $|+\rangle \equiv |m_{\mathrm{S}} = 0\rangle$, $|-\rangle \equiv |m_{\mathrm{S}} = -1\rangle$, $\boldsymbol{\nu}_+ \equiv \nu_0 \boldsymbol{e}_{\mathrm{z}}$, and $\boldsymbol{\nu}_- \equiv \nu_0 \boldsymbol{e}_{\mathrm{z}} + \boldsymbol{A}$. The evolution under the DD control is

$$\hat{U}_{\mathrm{DD}} = \left[\mathrm{e}^{-\mathrm{i}2\pi\hat{H}\tau}\mathrm{e}^{-\mathrm{i}\pi\hat{S}_{\mathrm{x}}}\mathrm{e}^{-\mathrm{i}2\pi\hat{H}\tau}\right]^{N_{\mathrm{p}}}, \tag{2}$$

where $\tau = t_{\mathrm{I}}/N_{\mathrm{p}}$. $\hat{U}_{\mathrm{DD}}$ can always be factorized to

$$\hat{U}_{\mathrm{DD}} = \mathrm{e}^{-\mathrm{i}\Phi\boldsymbol{n}\cdot\hat{\boldsymbol{I}}}\mathrm{e}^{\mathrm{i}2\alpha\hat{S}_{\mathrm{z}}\hat{I}_{\hat{\boldsymbol{\alpha}}}}. \tag{3}$$

### *2.2. First-order Magnus expansion for weakly coupled nuclear spins*

We write the evolution as $\hat{U}_{\mathrm{DD}} = \hat{U}_+|+\rangle\langle+| + \hat{U}_-|-\rangle\langle-|$ with

$$\begin{aligned}\hat{U}_+ &= (\mathrm{e}^{-\mathrm{i}\tau\pi\boldsymbol{\nu}_-\cdot\hat{\boldsymbol{I}}}\mathrm{e}^{-\mathrm{i}\tau\pi\boldsymbol{\nu}_+\hat{I}_{\mathrm{z}}})^{N_{\mathrm{p}}},\\ \hat{U}_- &= (\mathrm{e}^{-\mathrm{i}\tau\pi\boldsymbol{\nu}_+\cdot\hat{\boldsymbol{I}}}\mathrm{e}^{-\mathrm{i}\tau\pi\boldsymbol{\nu}_-\hat{I}_{\mathrm{z}}})^{N_{\mathrm{p}}}.\end{aligned} \tag{4}$$

Here, we consider the case of even pulse numbers. $\hat{U}_{\pm}$ can be expressed as a time-ordered integration

$$\hat{U}_{\pm} = \mathrm{T}\mathrm{e}^{-\mathrm{i}2\pi\int_0^{t_\mathrm{I}}\hat{H}_{\pm}(t)\mathrm{d}t}, \tag{5}$$

with

$$\hat{H}_{\pm}(t) = \hat{\bar{H}} \pm \frac{1}{2}\beta(t)\boldsymbol{A}\cdot\hat{\boldsymbol{I}}, \tag{6}$$

where $\hat{\bar{H}} = \bar{\boldsymbol{\nu}}\cdot\hat{\boldsymbol{I}}$, $\bar{\boldsymbol{\nu}} = \nu_0\boldsymbol{e}_\mathrm{Z} + \boldsymbol{A}/2$, and the modulation function $\beta(t)$ alternates between +1 and -1 every time a π-pulse is applied.

In the interaction picture defined by $\hat{\bar{H}}$, the evolution operator is

$$\hat{U}_\mathrm{DD} = \mathrm{e}^{-\mathrm{i}2\pi\bar{\boldsymbol{\nu}}\cdot\hat{\boldsymbol{I}}t_\mathrm{I}}\mathrm{T}\mathrm{e}^{-\mathrm{i}2\pi\hat{S}_z\int_0^{t_\mathrm{I}}\beta(t)\boldsymbol{A}\cdot\hat{\boldsymbol{I}}(t)\mathrm{d}t}, \tag{7}$$

where $\hat{\boldsymbol{I}}(t) = \mathrm{e}^{\mathrm{i}2\pi\bar{\boldsymbol{\nu}}\cdot\hat{\boldsymbol{I}}t_\mathrm{I}}\hat{\boldsymbol{I}}\mathrm{e}^{-\mathrm{i}2\pi\bar{\boldsymbol{\nu}}\cdot\hat{\boldsymbol{I}}t_\mathrm{I}}$. We decompose $\boldsymbol{A}$ to $\boldsymbol{A} = \boldsymbol{A}_\perp + \boldsymbol{A}_\mathrm{Z}$. The Z component part is averaged out and hence we obtain

$$U_\mathrm{DD} = \mathrm{e}^{-\mathrm{i}2\pi\bar{\boldsymbol{\nu}}\cdot\hat{\boldsymbol{I}}t_\mathrm{I}}\mathrm{e}^{-\mathrm{i}2\pi\hat{S}_z\int_0^{t_\mathrm{I}}\beta(t)\boldsymbol{A}_\perp\cdot\hat{\boldsymbol{I}}(t)\mathrm{d}t}. \tag{8}$$

The first order Magnus expansion, which is valid for weak hyperfine interaction, gives[5]

$$\mathrm{T}\mathrm{e}^{-\mathrm{i}2\pi\hat{S}_z\int_0^{t_\mathrm{I}}\beta(t)\boldsymbol{A}_\perp\cdot\hat{\boldsymbol{I}}(t)\mathrm{d}t} \approx \mathrm{e}^{-\mathrm{i}2\pi\hat{S}_z\int_0^{t_\mathrm{I}}\beta(t)\boldsymbol{A}_\perp\cdot\hat{\boldsymbol{I}}(t)\mathrm{d}t} \equiv \mathrm{e}^{2\mathrm{i}\alpha\hat{S}_z\boldsymbol{e}_\mathrm{X}\cdot\hat{\boldsymbol{I}}}, \tag{9}$$

where

$$\alpha = \frac{2A_\perp}{\bar{\nu}}\frac{\sin(N_\mathrm{p}\pi\bar{\nu}\tau)}{\cos(\pi\bar{\nu}\tau)}\sin^2\frac{\pi\bar{\nu}\tau}{2}, \tag{10}$$

and $\boldsymbol{e}_\mathrm{X}$ is the unit vector rotating from $\boldsymbol{A}_\perp$ by an angle $-\pi\bar{\nu}t_\mathrm{I}$ around the Z axis.

The readout period between the DD controls can be adjusted for fine tuning the nuclear spin precession. During the readout period, the perpendicular component of the hyperfine interaction $\boldsymbol{A}_\perp$ is averaged to zero by the fast precession of the nuclear spin (the Zeeman frequency ~MHz is much greater than the hyperfine interaction ~kHz in the experiment). The evolution of the nuclear spin during the readout period is $\hat{U}_\mathrm{read} = \exp(-\mathrm{i}2\pi\bar{\nu}t_\mathrm{read}\hat{I}_\mathrm{Z})$ plus a small dephasing along the Z axis (which results from the random population of the electron spin states, see Supplementary Note 4 below for more discussions). Since the dephasing about the Z axis is small and does

not affect the frequency of the nuclear spin precession in the X-Y plane, we can drop it in calculating the correlation function.

Therefore, the evolution of the electron spin and the nuclear spin, during the DD control and the readout period, can be written as

$$\hat{U}_T = \mathrm{e}^{-\mathrm{i}\Phi\hat{I}_\mathrm{Z}}\mathrm{e}^{2\mathrm{i}\alpha\hat{S}_\mathrm{z}\hat{I}_\mathrm{X}}, \tag{11}$$

a control phase gate plus a free precession by an angle $\Phi = 2\pi\bar{\nu}t_\mathrm{c}$ with hyperfine-modified frequency $\bar{\nu}$.

## Supplementary Note 3: Correlation functions for multiple nuclear spins

For multiple nuclear spins, the evolution operator is $\hat{U}_{\pm} = \prod_i \hat{U}_{i,\pm}$ if we neglect the interaction between the nuclear spins. Here $\hat{U}_{i,\pm} = \mathrm{e}^{-\mathrm{i}\Phi_i \hat{I}_{i,\mathrm{z}}} \mathrm{e}^{\pm\mathrm{i}\alpha_i \hat{I}_{i,\mathrm{x}}}$ is the unitary evolution operator of the *i*-th nuclear spin for the electron spin in the state $|\pm\rangle$. The Kraus operators of the multi-spin systems are $\hat{M}_{\pm} = \frac{\left(\prod_i e^{\mathrm{i}\alpha \hat{I}_{i,\mathrm{x}}} \pm \mathrm{i} \prod_i \mathrm{e}^{-\mathrm{i}\alpha_i \hat{I}_{i,\mathrm{x}}}\right)}{2}$. The precession is $\hat{U}[\hat{\rho}] = \left(\prod_i \hat{U}_i\right)[\hat{\rho}]$ with $\hat{U}_i[\hat{\rho}] \equiv \mathrm{e}^{-\mathrm{i}\Phi_i \hat{I}_{i,\mathrm{z}}} \hat{\rho} \mathrm{e}^{\mathrm{i}\Phi_i \hat{I}_{i,\mathrm{z}}}$. The correlation function is

$$C(N) \equiv \langle m_{k+N} m_k \rangle = \mathrm{Tr}\left[\hat{P}\left(\hat{U}\hat{M}\right)^{N-1} \hat{U}\hat{P}[\hat{\rho}_0]\right], \tag{12}$$

where the polarization operator $\hat{P}[\hat{\rho}] \equiv \hat{M}_+ \hat{\rho} \hat{M}_+^{\dagger} - \hat{M}_- \hat{\rho} \hat{M}_-^{\dagger}$ and $\hat{M}[\hat{\rho}] \equiv \hat{M}_+ \hat{\rho} \hat{M}_+^{\dagger} + \hat{M}_- \hat{\rho} \hat{M}_-^{\dagger}$. The initial state $\hat{\rho}_0$ is taken as unpolarized.

In general the weak measurement of the multiple nuclear spins via the projective measurement of a commonly coupled central electron spin can introduce many-body correlations, which is interesting but will not be studied here. Instead, we consider the case that the measurement strength is weak, i.e., $|\alpha_i| \ll 1$. In this case, we have $\hat{P}[\hat{\rho}] \approx \sum_i \alpha_i \hat{I}_{i,\mathrm{X}}$, which has no correlation in the leading order. Therefore, the following measurement induced dephasing and the precession can be considered as independent processes. The correlation function becomes a simple form as

$$C(N) \approx \sum_i C_i(N), \tag{13}$$

in which $C_i(N)$ is the correlation function of the sequential weak measurement if only the *i*-th nuclear spin is in the presence.

## Supplementary Note 4: Dephasing during the readout

During the readout period (the measurement cycle excluding the DD control), the rapid Larmor precession of the nuclear spin averages the perpendicular hyperfine interaction to be zero (during the resonant DD control, on the contrary, the perpendicular component is preserved), and the effective coupling becomes

$$H_{\mathrm{read}} = \bar{\nu}_0 \hat{I}_{\mathrm{Z}} + \sum_j |j\rangle\langle j| \otimes A_j \hat{I}_{\mathrm{Z}}, \tag{14}$$

where $|j\rangle$ denotes different levels (including the optically excited states and different charge states) of the NV centre and $A_j$ is the corresponding hyperfine constant. The second term in the r.h.s. of the equation above would induce dephasing of the nuclear spin along the *Z* axis. Depending on the correlation time $\tau_{\mathrm{c}}$ of the electron spin random jumps, the dephasing during the readout time ($t_{\mathrm{read}}$) is estimated to be

$$\gamma' \approx \begin{cases} 2\pi^2 A_{\mathrm{Z}}^2 t_{\mathrm{read}}{}^2 \text{ for } \tau_{\mathrm{c}} \gg t_{\mathrm{read}} (\text{inhomogeneous broadening}), \\ 4\pi^2 A_{\mathrm{Z}}^2 t_{\mathrm{read}} \tau_{\mathrm{c}} \text{ for } \tau_{\mathrm{c}} \ll t_{\mathrm{read}} \text{ (motional narrowing)}, \end{cases} \tag{15}$$

and in between for intermediate correlation times. The dephasing rate can be accounted by an effective interaction time $\tau_{\mathrm{eff}}$ and the dephasing rate can be written as $A_{\mathrm{Z}}^2 \tau_{\mathrm{eff}}^2$. In particular $\tau_{\mathrm{eff}} \approx 2\pi\sqrt{t_{\mathrm{read}}\tau_{\mathrm{c}}}$ in the motional narrowing regime. From the extrinsic broadening of C2 peak in Fig.4 of main text, the effective interaction time is estimated to be 600 μs for the case that electron is repetitively readout for 40 times. Correspondingly, the dephasing in the *Z* basis is $\gamma' = 1 \times 10^{-4}$, which is much smaller than $\gamma_{\mathrm{eff}} \approx 1 \times 10^{-3}$for C1.

## Supplementary Note 5: Data Processing

## *5. 1.* Reconstruction of correlation function from photon counts

The correlation function is $C(N)=\sum\limits_{m_k,m_{k+N}} m_k m_{k+N} p(m_k,m_{k+N})$, where $p(m_k,m_{k+N})$ is the joint probability for the two outputs under perfect projective measurements of the NV center. The correlation spectrum is obtained by Fourier transform $S(\nu)=\sum_{N=1}^{N_{\rm FT}}\exp(\mathrm{i}2\pi N\nu t_c)\,C(N)$.

### *5.1.1. Direct reconstruction*

The raw data is the photon number $n_k$ collected from each measurement cycle. The joint probability of the two outcomes $n_k$ and $n_{k+N}$ depends on the correlation $C(N)$ through

$$p(n_k,n_{k+N})=\sum_{m_k,m_{k+N}} D(n_k|m_k)D(n_{k+N}|m_{k+N})p(m_k,m_{k+N}), \tag{16}$$

where $D(n_k|m_k)$ is the probability of detecting $n_k$ photons given that the electron spin is in the state that would produce the output $m_k$ under a perfect projective measurement . Using the relations $p(+,+)=p(-,-)$ and $p(+,-)=p(-,+)$, we obtain $p(m_k,m_{k+N})=\sum\limits_{m_k,m_{k+N}}\frac{[1+m_k m_{k+N}C(N)]}{4}$ and hence the correlation of the photon counts

$$\begin{aligned}\langle n_k n_{k+N}\rangle &= \sum_{n_k,n_{k+N}} n_k n_{k+N} p(n_k,n_{k+N})\\ &=\frac{1}{4}\sum_{n_k,n_{k+N}} n_k n_{k+N}\sum_{m_k,m_{k+N}} D(n_{k+N}|m_{k+N})[1\\ &\quad + m_k m_{k+N}C(N)]\\ &=\bar{n}^2+\frac{1}{4}(\bar{n}_+-\bar{n}_-)^2C(N)\end{aligned} \tag{17}$$

where $\bar{n}_{\pm}=\sum_{n} nD(n|\pm)$ is the average photon counts for the NV center in the $\pm$ state, and $\bar{n}=\frac{(\bar{n}_{+}+\bar{n}_{-})}{2}$ is the average photon count. Thus the correlation is constructed from the photon count statistics as

$$C(N)=\frac{4(\langle n_k n_{k+N}\rangle-\bar{n}^2)}{(\bar{n}_{+}-\bar{n}_{-})^2}. \tag{18}$$

Now we estimate the fluctuation of the correlation as reconstructed from the photon statistics, which is subjected to the shot noises. Since $\bar{n}^2$ is a constant, its fluctuation has no contribution to the correlation spectrum at nonzero frequencies. The fluctuation of the denominator $(\bar{n}_{+}-\bar{n}_{-})^2$ would result in fluctuation of the overall amplitude of the correlation function and the spectrum without effects on the resonance frequency and width. Thus we only consider the fluctuation of the correlation $\langle n_k n_{k+N}\rangle$. For a finite sequence of $M$ outputs $\{n_1, n_2, \cdots, n_M\}$, the correlation is calculated by

$$\langle n_k n_{k+N}\rangle_M \approx \frac{\sum_{k=1}^{M-N} n_k n_{k+N}}{(M-N)}. \tag{19}$$

For $N \ll M$, the fluctuation $\delta\langle n_k n_{k+N}\rangle_M \approx \frac{\delta n_k n_{k+N}}{\sqrt{M}}$ according to the center limit theorem, where $\delta n_k n_{k+N}$ is the fluctuation of two joint outputs. By direct calculation we obtain

$$\begin{aligned}(n_k n_{k+N})^2 &= \frac{1}{4}\sum_{n_k, n_{k+N}} (n_k n_{k+N})^2 \sum_{m_k, m_{k+N}} D(n_k|m_k) D(n_{k+N}|m_{k+N})[1 \\ &\quad + m_k m_{k+N} C(N)] \\ &= \frac{1}{4}\left(\overline{n_{+}^2}+\overline{n_{-}^2}\right)^2+\frac{1}{4}\left(\overline{n_{+}^2}-\overline{n_{-}^2}\right) C(N)\end{aligned} \tag{20}$$

where $\overline{n_{\pm}^2} \equiv \sum_{n_k} n_k^2 D(n_k|\pm)$, which is $\overline{n_{\pm}^2}=\bar{n}_{\pm}^2+\bar{n}_{\pm}$ for Poisson distribution. Using the condition $C(N) \ll 1$, we obtain the fluctuation as

$$(\delta n_k n_{k+N})^2 = \overline{(n_k n_{k+N})^2} - \langle n_k n_{k+N}\rangle^2 = \left[\bar{n}^2 + \bar{n} + \frac{1}{4}(\bar{n}_+ - \bar{n}_-)^2\right]^2 - \bar{n}^4. \quad (21)$$

As a result, the noise amplitude becomes

$$\delta C(N) = \frac{\varepsilon_1}{\sqrt{M}} \quad (22)$$

with

$$\varepsilon_1 = \frac{4\sqrt{\left[\bar{n}^2+\bar{n}+\frac{(\bar{n}_+-\bar{n}_-)^2}{4}\right]^2 - \bar{n}^4}}{(\bar{n}_+-\bar{n}_-)^2}. \quad (23)$$

### *5.1.2 Reconstruction from single-shot readout*

When the NV center $^{14}$N nuclear spin is employed to assist readout of the NV center electron spin state (see Supplementary Note 1), the photon counts in each cycle of measurement can be quite large ($|\bar{n}_+ - \bar{n}_-| \gg 1$ ) and it is possible to choose a threshold photon count number $n_{\text{th}}$ between $n_-$ and $n_+$ such that the output is recorded as +1 or -1 if the photon counts in a cycle is above or below the threshold, i.e.,

$$s_k = \begin{cases} +1 & \text{for } n_k > n_{\text{th}}, \\ -1 & \text{for } n_k \le n_{\text{th}}. \end{cases} \quad (24)$$

For the NV center electron spin $m_k = \pm$ state, the conditional probability of output $s_k = \pm 1$ is

$$\begin{aligned} p_+ &\equiv p(+1|+) = \sum_{n_k > n_{\text{th}}} D(n_k|+) = 1 - p(-1|+), \\ p_- &\equiv p(-1|-) = \sum_{n_k < n_{\text{th}}} D(n_k|-) = 1 - p(+1|-). \end{aligned} \quad (25)$$

When $p_+ = p_- = 1$ , the photon detection constitutes a perfect single-shot measurement. The joint probability of two outputs is

$$p(s_k, s_{k+N}) = \sum_{m_k, m_{k+N}} p(s_k|m_k) p(s_{k+N}|m_{k+N}) p(m_k, m_{k+N}), \quad (26)$$

and correlation function is

$$\langle s_k s_{k+N}\rangle = \sum_{s_k, s_{k+N}} s_k s_{k+N} p(s_k, s_{k+N})$$

$$= \sum_{s_k, s_{k+N}} s_k s_{k+N} \sum_{m_k, m_{k+N}} p(s_k|m_k)p(s_{k+N}|m_{k+N})p(m_k, m_{k+N})$$

$$= \sum_{s_k, s_{k+N}} s_k s_{k+N} \sum_{m_k, m_{k+N}} p(s_k|m_k)p(s_{k+N}|m_{k+N}) \frac{1 + m_k m_{k+N} C(N)}{4}$$

$$= (p_+ - p_-)^2 + (p_+ + p_- - 1)^2 C(N). \tag{27}$$

Using $\langle s_k\rangle = \langle s_{k+N}\rangle = p_+ - p_-$, we reconstruct the correlation function of the weak measurements on the nuclear spin as

$$C(N) = \frac{\langle s_k s_{k+N}\rangle - \langle s_k\rangle\langle s_{k+N}\rangle}{(p_+ + p_- - 1)^2}. \tag{28}$$

With similar approaches in Supplementary note 5.1, the correlation of the output shot noises is

$$(\delta s_k s_{k+N})^2 = \langle s_k^2 s_{k+N}^2\rangle - \langle s_k s_{k+N}\rangle^2 \approx 1 - (p_+ - p_-)^4, \tag{29}$$

and the fluctuation of the correlation due to the photon shot noises

$$\delta C = \frac{\varepsilon_2}{\sqrt{M}}, \tag{30}$$

with

$$\varepsilon_2 = \frac{\sqrt{1 - (p_+ - p_-)^4}}{(p_+ + p_- - 1)^2}. \tag{31}$$

### *5.1.3. Error bar of Fourier transformation*

We denote $N_{\mathrm{FT}}$ time-domain data by a real vector $\mathbf{s} = \{C(1), C(2), \ldots, C(N_{\mathrm{FT}})\}$. The Fourier transform is

$$\boldsymbol{f} = \boldsymbol{U}\boldsymbol{s} \tag{32}$$

where $U_{ij} = \mathrm{e}^{-\mathrm{i}2(i-1)(j-1)\pi/N_{\mathrm{FT}}}$. The matrix has the properties $\boldsymbol{U}^{\mathrm{T}} = \boldsymbol{U}$ and $\boldsymbol{U}\boldsymbol{U}^{\dagger} = N_{\mathrm{FT}}$.

We assume that each elements of $\boldsymbol{s}$ has the normal distribution $\mathcal{N}(0, \sigma_{\mathrm{t}})$, where $\sigma_t$ denotes the noise amplitude in the time domain. We use $\boldsymbol{a} = \mathrm{Re}\mathbf{f}$ and $\boldsymbol{b} = \mathrm{Im}\mathbf{f}$ to denote the real part and imaginary part of $\boldsymbol{f}$. The covariance of $\boldsymbol{s}$ is

$$\langle \boldsymbol{s}\boldsymbol{s}^T \rangle = \sigma_{\mathrm{t}}^2 \mathbf{1}. \tag{33}$$

The covariance of the Fourier transform are

$$\begin{aligned} \langle \boldsymbol{a}\boldsymbol{a}^{\mathrm{T}} \rangle &= [(\mathrm{Re}\mathbf{U})]\langle \boldsymbol{s}\boldsymbol{s}^{\mathrm{T}} \rangle [(\mathrm{Re}\mathbf{U})]^{\mathrm{T}} \\ \langle \boldsymbol{b}\boldsymbol{b}^{\mathrm{T}} \rangle &= [(\mathrm{Im}\mathbf{U})]\langle \boldsymbol{s}\boldsymbol{s}^{\mathrm{T}} \rangle [(\mathrm{Im}\mathbf{U})]^{\mathrm{T}} \\ \langle \boldsymbol{a}\boldsymbol{b}^{\mathrm{T}} \rangle &= [(\mathrm{Re}\mathbf{U})]\langle \boldsymbol{s}\boldsymbol{s}^{\mathrm{T}} \rangle [(\mathrm{Im}\mathbf{U})]^{\mathrm{T}} \end{aligned} \tag{34}$$

After some simplification, the noise amplitude of real part and imaginary part is the same with each other and is linearly proportional to that of the time domain by the relation

$$\sigma_{\omega} = \sqrt{N_{\mathrm{FT}}/2}\sigma_{\mathrm{t}}, \tag{35}$$

independent of the frequency.

When processing the experimental data, what we fit is the absolute value of the Fourier transform (not its real part and its imaginary part). As a result, we should give the noise amplitude of the absolute value of the Fourier transform. The fluctuation of the absolute value is

$$\delta S = \begin{cases} \sigma_{\omega} & S \gg \sigma_{\omega} \\ \sqrt{2 - \pi/2}\,\sigma_{\omega} & S \ll \sigma_{\omega} \end{cases} \tag{36}$$

In the following, we neglect the irrelevant constant (because it is in the order of 1), we obtain the spectrum fluctuation due to photon shot noise as

$$\delta S \approx \frac{\varepsilon_{1,2}}{\sqrt{M}}\sqrt{N_{\mathrm{FT}}/2}. \tag{37}$$

The spectrum height at the resonance frequency is

$$S(\nu_{\mathrm{eff}}) \approx \frac{1}{2}\min(N_{\mathrm{C}}, N_{\mathrm{FT}}) \sin^2 \alpha, \tag{38}$$

where $N_{\mathrm{C}} \sim \gamma_{\mathrm{eff}}^{-1}$ is the life time of the correlation signal. To maximize the signal-to-noise ratio, we choose $N_{\mathrm{FT}} \approx N_{\mathrm{C}}$ for constructing the correlation spectrum from the experimental data .

Supplementary Figure 2 shows an example of the fluctuation of the correlation spectrum $|S(\nu)|$ (not its square), where the electron spin is repetitively read out for 40 times in each measurement cycle. The number of measurement cycles is about $M = 3 \times 10^7$. The averaged photon number collected per cycle is about $\bar{n} \approx 4.0$. The noise amplitude per cycle is $\varepsilon_1 \approx 28$. If we use $N_{\mathrm{FT}} = 1500$ time points for the Fourier transform, the noise amplitude of the spectrum is about $\delta S \approx 0.14$ and signal-to-noise ratio is about 10.6 for the highest peak as shown in Supplementary Fig. 2.

### *5.1.4. Comparison between the performance of the two methods*

We just need to compare the fluctuations of the output correlations $\varepsilon_1$ and $\varepsilon_2$ of the two methods in Supplementary Note 5.1.1 & 5.1.2. For the Poisson distribution of the photon counts $p(n|\pm) = \mathrm{e}^{-\bar{n}_\pm}\bar{n}_\pm^n/n!$, the optimal threshold photon counts for single-shot readout is

$$n_{\mathrm{th}} = \left|\frac{\bar{n}_+ - \bar{n}_-}{\ln\left(\frac{\bar{n}_+}{\bar{n}_-}\right)}\right|, \tag{39}$$

which is between $\bar{n}_+$ and $\bar{n}_-$.

If the averaged photon number is very small ($\bar{n}_{\pm} \ll 1$), the threshold $n_{\mathrm{th}} \approx 0$, so $p_+ = \bar{n}_+$ and $p_- = 1 - \bar{n}_-$. The fluctuations in the output correlations in this low photon count limit are

$$\varepsilon_1 \approx \frac{1}{\eta^2 \bar{n}}, \tag{40}$$

and

$$\varepsilon_2 \approx \frac{1}{\sqrt{2}\eta^2 \bar{n}^{3/2}}, \tag{41}$$

where $\eta = (\bar{n}_+ - \bar{n}_-)/(\bar{n}_+ + \bar{n}_-)$ is the contrast of the fluorescence. The reconstruction from the single-shot readout method has much larger fluctuation in the low photon count limit.

In the large photon count limit ($\bar{n} \gg 1$), $p_{\pm} \approx 1$. The fluctuations in the output correlations are

$$\varepsilon_1 \approx \sqrt{1 + 2\eta^{-2}}, \tag{42}$$

and

$$\varepsilon_2 \approx 1. \tag{43}$$

The single-shot readout approach would be much better when the contrast is small.

Supplementary Figure 3 (a) shows $\varepsilon_1$ (black line) and $\varepsilon_2$ (red line) as a function of $\bar{n}$ when the contrast is fixed to 0.18. As expected, $\varepsilon_2$ is larger than $\varepsilon_1$ when $\bar{n} \lesssim 0.5$ and the opposite when $\bar{n} \gtrsim 0.5$. We also show the ratio $\frac{\varepsilon_2}{\varepsilon_1}$ as a function of $\bar{n}$ in Supplementary Fig. 3 (b) for different contrasts. In all cases the single-shot readout scheme has larger fluctuation for $\bar{n} \lesssim 0.5$ and smaller fluctuations for $\bar{n} \gtrsim 0.5$. In our experiments, the largest $\bar{n}$ is about 4.0 when the electron state is repetitively read out via the auxiliary nitrogen nuclear spin (as for Fig. 4 in the main text). Under this condition, the single-shot readout approach has smaller fluctuations by a factor 2 than the direct reconstruction. Without repetitively readout, $\bar{n}$ is about

0.1 (as for Figs. 2 & 3 in the main text). Under such cases, the direct reconstruction approach has smaller fluctuations by a factor of 2.3 than the single-shot readout scheme. Since there is no orders of magnitude difference in fluctuations between the two methods, we use the direct reconstruction in all the cases.

### *5. 2.* Spatial sensing range

We consider the weak measurement limit for estimation of the sensing range.

The resonance signal of the correlation spectrum is

$$S\left(\nu_{\text{eff}}\right)\approx\frac{1}{2}N_{\text{C}}\sin^2\alpha\approx 2N_C A_{\perp}^2 t_{\text{I}}^2, \tag{44}$$

which is reached when the number of time points used for Fourier transform is $N_{\text{FT}}=N_{\text{C}}$. The correlation lifetime $N_{\text{C}}\approx 1/\left(\gamma_{\text{eff}}+\gamma_{\text{ex}}\right)$ (in units of measurement cycles), with the measurement induced dephasing per cycle $\gamma_{\text{eff}}=A_{\perp}^2 t_{\text{I}}^2$ and the dephasing during the waiting and readout periods $\gamma_{\text{ex}}\approx A_{\text{Z}}^2\tau_{\text{eff}}^2$. The noise amplitude in the frequency domain becomes

$$\delta S=\frac{\varepsilon_{1(2)}}{\sqrt{M}}\sqrt{N_{\text{C}}}, \tag{45}$$

where the fluctuation $\varepsilon_{1(2)}$ depending on the reconstruction method. The signal-to-noise ratio is

$$\text{SNR}=\frac{\sin^2\alpha}{2}\frac{\sqrt{MN_{\text{C}}}}{\varepsilon_{1(2)}}\approx\frac{2A_{\perp}^2 t_{\text{I}}^2}{\sqrt{A_{\perp}^2 t_{\text{I}}^2+A_{\text{Z}}^2\tau_{\text{eff}}^2}}\frac{\sqrt{M}}{\varepsilon_{1(2)}}. \tag{46}$$

Using the position dependence of the hyperfine interaction $A_{\text{Z}}=A_0\left(1-3\cos^2\theta\right)/d^3$ and $A_{\perp}=3A_0\cos\theta\sin\theta/d^3$ (for ${}^{13}C$ nuclear spin $A_0\approx -20\ \text{kHz}\cdot\text{nm}^3$), we obtain

the relation between signal-noise ratio and the spatial position of the target nuclear spin

$$\mathrm{SNR} = \frac{d_0^3}{d^3}\left|\sin(2\theta)\right| \Big/ \sqrt{1+\left(\frac{1+3\cos(2\theta)}{3\sin(2\theta)}\frac{\tau_{\mathrm{eff}}}{t_{\mathrm{I}}}\right)^2}, \tag{47}$$

where $d$ and $\theta$ is the distance from the central spin and polar angle, and

$$d_0 = \left(\frac{3A_0 t_{\mathrm{I}}\sqrt{M}}{\varepsilon_{1(2)}}\right)^{1/3}, \tag{48}$$

is the typical sensing distance. For the current parameters, the measurement times $M = 3\times10^7$ and $\varepsilon_1 = 28$ for 40 times repetitive readout. For the case of 100 pulse number ($t_{\mathrm{I}} \approx 18\mu\mathrm{s}$), we estimate that the typical detecting distance is $d_0 \approx 6\mathrm{nm}$. Supplementary Figure 4 shows some examples of the spatial range of sensing for various interaction times.

The ultimate sensing range is limited by the coherence time of the NV center ($t_{\mathrm{I}} \le T_2$) and the photon-shot noise $\varepsilon_{1(2)} \ge 1$. For an NV center in bulk diamond with coherence time ~ 1 ms, $d_0^{\max} = (3A_0T_2)^{1/3} M^{1/6} \approx 4M^{1/6}$ (nm). For a shallow NV center located about 8 nm below diamond surface, the coherence time can reach about 100 μs [6], the corresponding sensing range would be about 16 nm. For a hydrogen nuclear spin with distance 8 nm away from such an NV center, the coupling is about 0.15 kHz. The corresponding spectral resolution is estimated to be 0.3 Hz.

## *5.3. Correction of systematic errors*

The systematic errors that affect the NV center fluorescence, including slow spatial drift of the NV center out of the microscope focus and oscillation of the laser output power due to the power grid will be reflected in the correlation function. We correct

these effects by fitting and subtracting an exponential decay, which results from the slow focus drift, and also fit and subtract a 100 Hz oscillation of the form, which stems from a 100 Hz modulation of the diode laser output power, due to the rectified 50 Hz AC power grid.

### *5.4. Determination of hyperfine interaction*

The hyperfine interaction $A_Z$ is determined by $\Phi = (\nu_0 + A_Z/2)t_c \bmod 2\pi$, which has ambiguity in multiples of $2\pi$. Further ambiguity is caused by the symmetry of the correlation function under the transformation $\Phi \leftrightarrow -\Phi \bmod 2\pi$.The first kind of ambiguity is removed as long as the hyperfine-renormalized Larmor frequency has been roughly determined with precision better than $1/t_c$ (e.g., by the resonant condition of DD control). The second kind of ambiguity can be removed by varying $t_c$, which shift the resonance peaks of $\Phi$ and $2\pi - \Phi$ toward opposite directions in frequency. The correlation spectrum $|S(\nu)|$ (not its square) is shown in Supplementary Fig. 5 Among all the peaks around $\nu - \nu_0 \approx 0.572\text{kHz}$, the common peak for all values of $t_c$ (indicated by the red arrow) is the resonance peak, which yields $\bar{\nu} = \nu_0 + A_Z/2$ and hence $A_Z = 1.144$ kHz with error 54 Hz.

### *5.5. Determine the error bar of the parameters*

For a set of data $\{\nu_i, f_i\}, i = 1, \cdot\cdot, N_{\text{FT}}$ as the signal (for example, the signal in the frequency domain), we fit it by a theoretical function $f(\nu, \boldsymbol{\lambda})$ with parameters being $\boldsymbol{\lambda}$. We assume that the signal $f_i$ obeys a Gaussian distribution $\text{N}(0, \sigma_i)$. According to the Bayesian formula, the distribution of fitting parameters $\boldsymbol{\lambda}$ conditioned on the signal now becomes

$$P(\boldsymbol{\lambda}|\mathbf{x}) \propto \exp\{-\Delta(\boldsymbol{\lambda})\}, \tag{49}$$

where

$$\Delta(\boldsymbol{\lambda}) = \sum_{i=1}^{N_{\mathrm{FT}}} \frac{\left[f_i - f(v_i, \boldsymbol{\lambda})\right]^2}{2\sigma_i^2} \tag{50}$$

is the cost function. The optimized estimation of $\boldsymbol{\lambda}$ is $\boldsymbol{\lambda}_{\mathrm{e}}$ which maximizes the distribution $P(\boldsymbol{\lambda}|\mathbf{x})$, or minimize $\Delta(\boldsymbol{\lambda})$ with

$$\frac{\partial \Delta(\boldsymbol{\lambda})}{\partial \boldsymbol{\lambda}}\Big|_{\boldsymbol{\lambda}=\boldsymbol{\lambda}_{\mathrm{e}}} = 0. \tag{51}$$

Around the peak, the distribution can be expanded as

$$P(\boldsymbol{\lambda}|\mathbf{x}) \propto \exp\left\{-\frac{1}{2}(\boldsymbol{\lambda}-\boldsymbol{\lambda}_{\mathrm{e}})^T g\boldsymbol{\Sigma} g(\boldsymbol{\lambda}-\boldsymbol{\lambda}_{\mathrm{e}})\right\}, \tag{52}$$

where

$$\boldsymbol{\Sigma} = \frac{\partial}{\partial \boldsymbol{\lambda}}\left(\frac{\partial \Delta(\boldsymbol{\lambda})}{\partial \boldsymbol{\lambda}}\right)\Bigg|_{\boldsymbol{\lambda}=\boldsymbol{\lambda}_{\mathrm{e}}} \tag{53}$$

is a matrix. As a result, the covariance matrix

$$\langle(\boldsymbol{\lambda}-\boldsymbol{\lambda}_{\mathrm{e}})(\boldsymbol{\lambda}-\boldsymbol{\lambda}_{\mathrm{e}})\rangle = \boldsymbol{\Sigma}^{-1} \tag{54}$$

and the error bar of each parameters is

$$\delta\lambda_i = 2\sqrt{\left(\boldsymbol{\Sigma}^{-1}\right)_{ii}} \tag{55}$$

As an example, we fit the peak in Supplementary Fig. 5. Here, the fitting curve is

$$f(v, \boldsymbol{\lambda}) = A\sqrt{\frac{\gamma^2}{(x-x_0)^2+\gamma^2}} \tag{56}$$

and the fitting parameters is $\boldsymbol{\lambda} = \{A, x_0, \gamma\}$ . $\sigma_i = \varepsilon_1\sqrt{N_{\mathrm{FT}}/(2M)}$ is the noise amplitude in the frequency domain. For the XY8 case, $M = 1.9\times10^{10}$. We choose $N_{\mathrm{FT}} = 2000$ and hence $\sigma_i = 0.08$. Using the above theory, the parameter is fitted to

$x_0 = 0.572\text{kHz} \pm 27\text{Hz}$ and $\gamma = 73\text{Hz} \pm 47\text{Hz}$, where $x_0$ is the estimation of the difference $\bar{v} - v_0$ between the precession frequency and the bare Larmor frequency. The error bars in the figures of the main text are obtained similarly.

### *5.6. High-precision measurement of the bare Larmor frequency*

To determine the strength of the longitude hyperfine coupling, the bare Larmor frequency of the $^{13}$C nuclear spins is determined with high precision using the method in Ref. [1]. Since this measurement relies on a correlation spectroscopy scheme, where free evolution of the detected spins occurs, while the NV electron spin is initialized into the $m_S = 0$ state, no hyperfine coupling is visible in the resulting spectrum. The method measures the difference between the Larmor frequency and the frequency of an inductive radio-frequency wave with $v_{\text{in}} = 2.730133\ \text{MHz}$. The difference is measured to be $v_0 - v_{\text{in}} = 10.0005\ \text{kHz} \pm 0.39\ \text{Hz}$ (Fig. S6). As a result, the bare Larmor frequency is $v_0 = 2.740134\ \text{MHz} \pm 0.39\ \text{Hz}$.

### *5.7. High resolution spectroscopy by NV2*

The high resolution spectroscopy in Fig. 4 of the main text is performed on NV2 instead of NV1 (which is measured for Figs. 2 & 3 of the main text). The measurement induced back action on the target nuclear spin is very small because the target is relatively far away from the central spin. This experiment has been carried out in two runs, generating two sets of data, Data1 and Data2. The electron spin is read out repeatedly for 40 times in Data1 and both for 40 times and 80 times in Data2. The fluctuations of the correlation due to the photon shot noises are estimated to be

$\varepsilon_1 = 28$ and $\varepsilon_1 = 20$ for case that electron is repetitively readout for 40 times and 80 times, respectively.

For Data1, the bare Larmor frequency of the nuclear spins has been measured very precisely with the method in Ref. [1]. The correlation spectrum is shown in Supplementary Fig. 7. Two nuclear spins are resolved. As the measurement strength is increased by applying more DD pulses, the peak width is broadened and more nuclear spins become visible. In Supplementary Fig. 7(c), four nuclear spins are detected. We concentrate on studying the C1 and C2 nuclear spins since these two nuclear spins are detected in all three cases of Supplementary Fig. 7.

The correlation spectrum from Data2 is shown in Supplementary Fig. 8. Supplementary Figure 8 (a-c) presents the correlation spectrum when electron state is repetitively read out for 40 times. The peaks in Supplementary Fig. 7 are also found in Supplementary Fig. 8. Supplementary Figure 8 (d-f) presents the correlation spectrum when the electron state is repetitively read out for 80 times. In Supplementary Fig. 8 (f), the C2 peak disappears, which is ascribed to the coherent trapping effect. For $t_c = 322.62400$ μs as chosen in this case, $|\sin\Phi| \approx 0.06$ for peak C2 is very close to $\tan^2(\alpha/2) \approx 0.05$ (for the estimated value $A_\perp \approx 4.02$ kHz in the main text). As there is some uncertainty in estimating $A_\perp$, the real value of $|\sin\Phi|$ may be smaller than $\tan^2(\alpha/2)$. As a result, the spin C2 is coherently trapped and its resonance is pinned at zero frequency (relative to $\nu_0$), which is not observed in the spectra since the static background has been subtracted from the correlation function in our data processing.

In comparison with the spectra from Data1, the peaks in the spectra from Data2 are shifted overall by about 40Hz. This shift comes from the shift of the magnetic field because between the two runs of experiments the crystal was moved inside the

slightly inhomogeneous magnetic field. In Supplementary Fig. 9 (a) and (b), we plot the peak positions of C1 and C2 for the two data sets. The shifts of C1 and C2 are both around 40Hz. We also plot the frequency difference between C1 and C2 for these two data sets. The difference is nearly unchanged both for different pulse numbers and different data sets. From the shift of the resonances from Data1 to Data2, the Larmor frequency for the Data2 is calibrated to $\nu_0' = 2.740090\ \mathrm{MHz} \pm 7.9\ \mathrm{Hz}$. The calibrated Larmor frequency is used in the correlation spectra shown in Fig. 4 of the main text.

## Supplementary Note 6: Data acquisition time

We neglect the background decoherence of the nuclear spins. Under this condition, the correlation of sequential weak measurements has the form

$$C(N) = 8\pi\Delta\nu t_{\mathrm{c}} \mathrm{e}^{-N2\pi\Delta\nu t_c} \cos 2N\pi \nu_{\mathrm{eff}} t_{\mathrm{c}}, \tag{57}$$

where $\Delta\nu = \sin^2\alpha / (8\pi t_{\mathrm{c}})$ is the resolution of the frequency and $\alpha$ quantifies the measurement strength. If we use $N_{\mathrm{FT}}$ data points to perform the Fourier transform, the optimized peak signal saturates to its maximum

$$S(\nu_{\mathrm{eff}}) = 2, \tag{58}$$

when $N_{\mathrm{FT}} \approx 1/(2\pi\Delta\nu t_{\mathrm{c}})$. The noise amplitude for the spectrum is

$$\delta S = \varepsilon_{1,2}\sqrt{\frac{N_{\mathrm{FT}}}{2M}} = \varepsilon_{1,2}\sqrt{\frac{1}{4\pi\Delta\nu T^{\mathrm{D}}}}, \tag{59}$$

where the data acquisition time $T^{\mathrm{D}} = Mt_{\mathrm{c}}$. For a given signal-to-noise ratio $\mathrm{SNR} \equiv S(\nu_{\mathrm{eff}}) / \delta S$ the data acquisition time to achieve a given resolution is thus

$$T^{\mathrm{D}} = \mathrm{SNR}^2 \frac{\varepsilon_{1,2}^2}{16\pi\Delta\nu}. \tag{60}$$

***Data acquisition time for the Ramsey scheme***

The Ramsey protocol begins with a measurement on the target though a DD sequence (with duration $t_{\mathrm{I}}$) and electron spin measurement, then a free precession time is inserted, and finally another measurement is implemented. The precession time *t* is swept from 0 to $T$ with a step $\tau$. For each precession time, the protocol is repeated for $M$ times to obtain the correlation between the two measurements. The final signal is the Fourier transform of the correlation.

Since there is no measurement in the precession time, there is no back-action and hence the correlation signal has the form

$$C(t) = 4\sin^2 \alpha_{\mathrm{R}} \cos 2\pi \nu_{\mathrm{eff}} t \,, \tag{61}$$

$\alpha_{\mathrm{R}} = 2A_{\perp} t_{\mathrm{I}}$ quantifies the measurement strength in the DD process. In the following, we estimate the data acquisition time for achieving a given resolution.

The discrete Fourier transform gives

$$S(\nu_j) = 4\sin^2 \alpha_{\mathrm{R}} \sum_{n=1}^{T/\tau} \cos(2\pi n \nu_{\mathrm{eff}} \tau) \mathrm{e}^{2\pi \mathrm{i} n \nu_j \tau} = 2\sin^2 \alpha_{\mathrm{R}} \left[ \frac{1-\mathrm{e}^{\mathrm{i}2\pi(\nu_j+\nu_{\mathrm{eff}})T}}{1-\mathrm{e}^{\mathrm{i}2\pi(\nu_j+\nu_{\mathrm{eff}})\tau}} + \frac{1-\mathrm{e}^{-\mathrm{i}2\pi(\nu_j-\nu_{\mathrm{eff}})T}}{1-\mathrm{e}^{\mathrm{i}2\pi(\nu_j-\nu_{\mathrm{eff}})\tau}} \right] \tag{62}$$

where $\nu_j = (j-1)/T$. The peak signal of the FFT is given by

$$S(\nu_{\mathrm{eff}}) \approx 2\frac{T}{\tau} \sin^2 \alpha_{\mathrm{R}} \,. \tag{63}$$

The noise amplitude is

$$\delta S = \frac{\varepsilon_{1,2}}{\sqrt{M}} \sqrt{\frac{T}{2\tau}} \,, \tag{64}$$

with $\varepsilon_{1,2} / \sqrt{M}$ giving the noise amplitude of each data in the time domain. The signal-to-noise ratio becomes

$$\mathrm{SNR} = \frac{S(\nu_{\mathrm{eff}})}{\delta S} = \frac{4\sin^2 \alpha_{\mathrm{R}}}{\varepsilon_{1,2}} \sqrt{\frac{T^D}{T}} = \frac{4\sin^2 \alpha_{\mathrm{R}}}{\varepsilon_{1,2}} \sqrt{2\pi T^{\mathrm{D}} \Delta\nu} \tag{65}$$

where $T^{\mathrm{D}} = MT^2 / (2\tau)$ is the total data acquisition time and $\Delta\nu = 1/(2\pi T)$ is the spectrum resolution.

As a result, the data acquisition time for achieving a given resolution $\Delta\nu$ and signal-to-noise ratio SNR becomes

$$T^{\mathrm{D}} = \frac{1}{2\sin^4 \alpha_{\mathrm{R}}} \left( \mathrm{SNR}^2 \frac{\varepsilon_{1,2}^2}{16\pi\Delta\nu} \right), \tag{66}$$

which has an extra factor $1/(2\sin^4 \alpha_R)$ in comparison with the data acquisition time for the sequential weak measurement protocol.

Since no measurement is performed in the precessing process, the measurement back action on the target nuclear spin is absence. Hence, one can maximize the measurement strength by choosing proper DD duration $t_{\mathrm{I}}$. The final result is

$$T^{\mathrm{D}} = \left( \mathrm{SNR}^2 \frac{\varepsilon_{1,2}^2}{16\pi\Delta\nu} \right) \times \begin{cases} \frac{1}{2}, \text{if } A_{\perp} > A_c, \\ \frac{1}{2}\left( \frac{A_c}{A_{\perp}} \right)^4, \text{if } A_{\perp} < A_c, \end{cases} \tag{67}$$

where $A_c = 1/(2T_1)$ and $T_1$ is the life time of electron spin. For sensing a weakly coupled nuclear spin, the Ramsey protocol requires a data acquisition time longer by a factor of : $1/(2A_{\perp}T_1)^4$ than the sequential weak measurement method.

***Data acquisition time for the Ramsey method enhanced with repetitive measurement***

In the above discussion of the Ramsey scheme, the quantum measurement of the target spin initialization and readout is assumed to be weak for nuclear spins coupled weaker than $A_c = 1/(2T_1)$. This assumption results in a long data acquisition time of the technique for weakly coupled spins. By repeating $N_{\mathrm{rep}}$ times the weak measurement for each free evolution, the initialization and readout fidelities and therefore the obtained information by the Ramsey measurement can be increased[7,8]. To estimate the data acquisition time considering this enhancement, the finite time of each shot of

measurement needs to be considered, which was previously neglected.

We assume that each measurement unit cost time $t_I$ and the measurement unit is repeated for $N_{\text{rep}}$ times to enhance the signal. The equations for the signal and its deviation become

$$S = N_{\text{rep}} \sin^2 \alpha \frac{2T}{\tau}, \tag{68}$$

$$\delta S = \frac{\sqrt{N_{\text{rep}}}\epsilon_{1,2}}{\sqrt{M}} \sqrt{\frac{T}{2\tau}}. \tag{69}$$

The number of measurements $M$ within a given accumulation time $T^{\text{D}}$ becomes

$$M = \frac{2\tau T^D}{T^2 + T N_{\text{rep}} t_{\text{I}}}. \tag{70}$$

The SNR is

$$\text{SNR} = \frac{4 \sin^2 \alpha}{\epsilon_{1,2}} \sqrt{\frac{N_{\text{rep}} T}{\tau M}} = \frac{4 \sin^2 \alpha}{\varepsilon_{1,\ 2}} \sqrt{\frac{2 N_{\text{rep}} T T^{\text{D}}}{T^2 + T N_{\text{rep}} t_{\text{I}}}}. \tag{71}$$

To achieve the spectral resolution $\Delta\nu = \frac{1}{2\pi T}$, the acquisition time is

$$T^{\text{D}} = \text{SNR}^2 \frac{\epsilon_{1,2}^2}{32 N_{\text{rep}} \sin^4 \alpha} \left( \frac{1}{2\pi \Delta\nu} + N_{\text{rep}} t_{\text{I}} \right). \tag{72}$$

For distant nuclear spin $A_\perp T_1 \ll 1$, the measurement strength is $\sin\alpha \approx 2 A_\perp T_1 \ll 1$ and duration $t_{\text{I}}$ can be optimized to $T_1$ to enhance the measurement strength. Hence, the acquisition time is optimized to

$$T^{\text{D}} = \text{SNR}^2 \frac{\epsilon_{1,2}^2}{16\pi\Delta\nu} \frac{1}{4 N_{\text{rep}} (2 A_\perp T_1)^4} \left( 1 + N_{\text{rep}} 2\pi \Delta\nu T_1 \right). \tag{73}$$

We then optimize the repetitive times, we obtain optimized acquisition time

$$T^{\text{D}} = \text{SNR}^2 \frac{\epsilon_{1,2}^2}{16\pi\Delta\nu} \frac{\pi \Delta\nu T_1}{2 (2 A_\perp T_1)^4}. \tag{74}$$

The desired resolution depends on the hyperfine coupling. For a rough estimation, $\Delta\nu \sim 0.1 \cdot A_\perp$may be a proper value. In such a case, we obtain

$$T^D = \left(\mathrm{SNR}^2 \frac{\epsilon_{1,2}^2}{16\pi\Delta\nu}\right)\frac{\pi}{4(2A_{\perp}T_1)^3}. \tag{75}$$

This data acquisition time is longer than that required for the weak measurement protocol to achieve the same spectral resolution by a factor of $\pi/[4(2A_{\perp}T_1)^3]$. For a rough estimation, if $A_{\perp} \approx 50\mathrm{Hz}$, the ratio is about 785.

## Supplementary References


1. Pfender, M. *et al.* Nonvolatile nuclear spin memory enables sensor-unlimited nanoscale spectroscopy of small spin clusters. *Nat. Commun.* **8**, 834 (2017).

2. Neumann, P. *et al.* Single-Shot Readout of a Single Nuclear Spin. *Science* **329**, 542–544 (2010).

3. Jiang, L. *et al.* Repetitive Readout of a Single Electronic Spin via Quantum Logic with Nuclear Spin Ancillae. *Science* **326**, 267–272 (2009).

4. Aslam, N., Waldherr, G., Neumann, P., Jelezko, F. & Wrachtrup, J. Photo-induced ionization dynamics of the nitrogen vacancy defect in diamond investigated by single-shot charge state detection. *New J. Phys.* **15**, 013064 (2013).

5. Ma, W.-L. & Liu, R.-B. Angstrom-Resolution Magnetic Resonance Imaging of Single Molecules via Wave-Function Fingerprints of Nuclear Spins. *Phys. Rev. Appl.* **6**, 024019 (2016).

6. Myers, B. A. *et al.* Probing Surface Noise with Depth-Calibrated Spins in Diamond. *Phys. Rev. Lett.* **113**, 027602 (2014).

7. Gefen, T., Khodas, M., McGuinness, L. P., Jelezko, F. & Retzker, A. Quantum spectroscopy of single spins assisted by a classical clock. *Phys. Rev. A* **98**, 013844 (2018).

8. Liu, G.-Q. *et al.* Single-Shot Readout of a Nuclear Spin Weakly Coupled to a Nitrogen-Vacancy Center at Room Temperature. *Phys. Rev. Lett.* **118**, 150504 (2017).
